# STARD 2015 was reproducible in a large set of studies on glaucoma

**DOI:** 10.1371/journal.pone.0186209

**Published:** 2017-10-12

**Authors:** Gianni Virgili, Manuele Michelessi, Alba Miele, Francesco Oddone, Giada Crescioli, Valeria Fameli, Ersilia Lucenteforte

**Affiliations:** 1 Department of Translational Surgery and Medicine, University of Florence, Florence, Italy; 2 IRCS Fondazione G.B. Bietti, Rome, Italy; 3 Department of Neurosciences, Psychology, Drug Research and Child Health (NEUROFARBA), University of Florence, Florence, Italy; 4 Ophthalmology unit, Department of Sens, Organs, University of Rome “Sapienza”, Rome, Italy; TNO, NETHERLANDS

## Abstract

**Aim:**

To investigate the reproducibility of the updated Standards for the Reporting of Diagnostic Accuracy Studies tool (STARD 2015) in a set of 106 studies included in a Cochrane diagnostic test accuracy (DTA) systematic review of imaging tests for diagnosing manifest glaucoma.

**Methods:**

One senior rater with DTA methodological and clinical expertise used STARD 2015 on all studies, and each of three raters with different training profiles assessed about a third of the studies.

**Results:**

Raw agreement was very good or almost perfect between the senior rater and an ophthalmology resident with DTA methods training, acceptable with a clinical rater with little DTA methods training, and only moderate with a pharmacology researcher with general, but not DTA, systematic review training and no clinical expertise. The relationship between adherence with STARD 2015 and methodological quality with QUADAS 2 was only partial and difficult to investigate, suggesting that raters used substantial context knowledge in risk of bias assessment.

**Conclusions:**

STARD 2015 proved to be reproducible in this specific research field, provided that both clinical and DTA methodological expertise are achieved through training of its users.

## Introduction

Several methodological tools have been introduced to make biomedical research more transparent, more reproducible and of better quality. [1,2] The Standards for the Reporting of Diagnostic Accuracy Studies (STARD) checklist [3] has been widely used to assess the reporting of diagnostic test accuracy (DTA) studies, often finding modest adherence with STARD recommendations. [4,5] The Quality Assessment of Diagnostic Accuracy Studies (QUADAS) [6] tool is a multi-domain checklist recommended by the Cochrane and by the U.K. National Institute for Health and Clinical Excellence to be used in systematic reviews for assessing the methodological quality and risk of bias of DTA studies. In 2011 a revised and improved version of QUADAS was published (QUADAS 2). [7]

Glaucoma is a common chronic ocular disease leading to slowly progressive peripheral visual field loss due to optic nerve damage [8]. Its early diagnosis may be challenging but still essential since visual field loss cannot be reversed and treatment only aims to reduce progression [9,10]. Diagnostics tests that are objective and reliable can be used in primary care to improve glaucoma diagnosis [8,9]. Tests which investigate retinal nerve fiber layer (RNFL) thickness have the advantage of providing objective and reliable anatomic measures, and have been proposed as triage tests for patients referred from primary eye care [11].

In the current study we investigated the application of the updated version of STARD (STARD 2015) [12] using a large set of studies that were included in a Cochrane review on RNFL and optic nerve head (ONH) imaging tests for diagnosing manifest glaucoma. [13] Previous research has shown only modest adherence with the original STARD version of DTA glaucoma studies. [14–17] In order to assess the adherence of studies published in this research field with the updated version of STARD, we needed to check whether judgements made using STARD 2015 are reproducible. Our aim was to assess inter-observer agreement and its determinants, as well as the relationship between STARD 2015 and QUADAS 2 score. Moreover, we investigated how completeness of reporting assessed using STARD 2015 was correlated with study methodological quality using QUADAS 2.

## Materials and methods

We considered 106 studies included in a Cochrane DTA systematic review published in 2016, which aimed to evaluate the accuracy of the latest version of three imaging devices for diagnosing manifest glaucoma: optical coherence tomography (OCT), Heidelberg retinal tomography (HRT) or scanning laser polarimetry (GDx). [13] These tests may help clinicians to identify structural damage at the level of the RNFL and ONH that can be used for an objective diagnosis of manifest glaucoma diagnosed by clinical assessment of the visual field and ONH.

Although STARD 2015 was not available when most of these studies were published, this tool was used to expand on previous studies in this field [14–17] in order to inform readers and researchers on the current status of this research and set a starting point for future investigations. The STARD 2015 checklist comprises 30 items, covering 6 domains (Title and abstract, Introduction, Methods, Results, Discussion, Other information). Four items (10, 12, 13 and 21) comprise two sub-items (a and b), one typically referring to an index test and one to a reference standard test. [12]

The STARD checklist was developed to be applied to all types of medical tests, disease or different disciplines to make its application simpler for authors. We selected four raters to investigate agreement: a senior DTA researcher and glaucoma specialist (MM), who rated all 106 studies, and three junior raters who assessed one third of the articles each: a glaucoma specialist with little specific DTA training (VF), an ophthalmology resident with DTA research and glaucoma training (AM) and a pharmacology researcher with experience in intervention, but not DTA, systematic reviews and no clinical experience (GC). Discrepancies between raters were adjudicated by discussion with a senior clinician with DTA methods expertise (GV). To adapt the checklist to the specific test/disease considered in the included studies of this review, we first prepared guidance criteria and piloted them on five studies. After the pilot, we drafted the final guidance criteria which did not include item 2 (structured abstract) because a new checklist for abstracts is being prepared, item 13a (availability of clinical information) and item 25 (test-related adverse events) as they were not applicable to our index tests. A total of 31 items were assessed by two raters as explained above. For each study, each item was scored as “yes” or “no”. Therefore, for each item we calculated the percentage of studies scored “yes” as the measure of adherence with STARD and identified common patterns of disagreement apart from insufficient exploration of the article text.

We investigated whether adherence with STARD 2015 is associated with better methodological quality with QUADAS 2 as follows. We considered that specific STARD domains may influence the ability to rate signaling questions that guide the judgement on QUADAS 2 risk of bias domains. Specifically, STARD 2015 items 6, 7, 8, 9 and 20, 21a and 21b concerned QUADAS 2 Patient selection domain, items 10a, 12a and 13a concerned the Index test domain, items 10b, 11, 12b and 13b concerned the Reference standard domain, and items 19, 20, 21 and 22 concerned the Flow and Timing domain.

We chose to report raw agreement since several STARD 2015 items were rated yes or no for almost all studies, which makes Cohen’s kappa shrink towards nil even in the presence of substantial raw agreement. Inference on raw agreement was made considering that 50% agreement is found simply by chance with dichotomous data, such as in our case. Cohen’s kappa was calculated only as an overall measure across all items for pairs of raters, thus ignoring repeated measures i.e. the assessment of multiple features at the study level. We used mixed logistic models with disagreement between the senior rater and any other rater as a response variable to investigate the effect of potential determinants (i.e. impact factor and publication year) on agreement, separately for each STARD 2015 item. In these models studies were a random effect, publication year was a assumed to have a logit-linear effect. For impact factor approximate tertiles were used and the lowest vs. highest tertiles were contrasted. Analyses were conducted using Stata 14.2 software (StataCorp, College Station, TX).

## Results

### Characteristics of included studies

Readers can refer to Michelessi 2016 for details on the included studies. [13] In short, we included 106 studies investigating one (n = 94) or more (n = 12) imaging tests among OCT, GDx and HRT; only more recent device versions were considered. The reference standard was combined functional (visual field, VF) and anatomic (clinical ONH examination) verification in 67 studies; the remaining 37 studies relied on either VF damage only (29 studies) or ONH/RNFL damage only (10 studies) as criteria for confirming glaucoma.

Michelessi et al.^9^ assessed the methodological quality of the studies using the QUADAS 2 checklist. The main quality issue was the use of a two-group (case-control) design (103 studies) or an unclear study design (two studies) in nearly all studies. This design is known to overestimate accuracy because it can increase the difference of the trait under investigation (RNFL thickness or ONH morphology) in patients with or without glaucoma, especially if healthy controls are used. [18]

Several RNFL or ONH parameters were compared in each study and all study authors compared sensitivity at fixed specificities, usually at 0.90 or 0.95. The reference standard was rated as good when VF only was used to detect the presence of glaucoma (27 studies) because the patient's function is affected and because VF explores a different dimension compared to that assessed by ONH/RNFL imaging tests. Masking reference test classification to index test results was often unclear (75 studies) or not adopted (one study); only 30 studies reported a masked interpretation with respect to index test results.

With regard to QUADAS 2 Flow and Timing domain, exclusions were generally due to poor-quality images, which we considered a good quality criterion for the assessment of the Index test domain.

### Inter-rater agreement

Overall raw agreement between the senior rater and any other rater was good: 90% or more for 15 items (48.4%); 80% to 89% for 10 items (32.3%); agreement was moderate at 62% to 75% for 6 items (19.4%) (Table 1).

**Table 1:** Interrater agreement between the senior rater and any other rater (Overall Agreement) or each of three other raters (Ophthalmology resident, Ophthalmologist, Pharmacology researcher). The joint judgement is presented as Positive Reporting.

	No	Item	Positive Reporting, N (%)	Overall Agreement, N (%)	Ophthalmology resident, N (%)	Ophthalmologist, N (%)	Pharmacology researcher, N(%)
**TITLE OR ABSTRACT**							
	**1**	Identification as a study of diagnostic accuracy using at least one measure of accuracy (such as sensitivity, specificity, predictive values, or AUC)	106 (100.00)	101 (95.3)	34 (97.1)	33 (94.3)	35 (94.59)
**INTRODUCTION**							
	**2**	Structured summary of study design, methods, results, and conclusions (for specific guidance, see STARD for abstracts)	Not applicable				
	**3**	Scientific and clinical background, including the intended use and clinical role of the index test	10 (9.4)	67 (63.21)	34 (97.1)	21 (60)	12 (33.3)
	**4**	Study objectives and hypotheses	40 (37.4)	87 (82.08)	35 (100.00)	23 (65.7)	29 (80.1)
**METHODS**							
Study design	**5**	Whether data collection was planned before the index test and reference standard were performed (prospective study) or after (retrospective study)	59 (55.7)	89 (84)	32 (91.4)	32 (91.4)	25 (69.4)
Participants	**6**	Eligibility criteria	103 (97.2)	97 (91.5)	34 (97.1)	33 (94.3)	30 (83.3)
	**7**	On what basis potentially eligible participants were identified (such as symptoms, results from previous tests, inclusion in registry)	53 (50)	77 (72.6)	32 (91.4)	28 (77.1)	18 (50)
	**8**	Where and when potentially eligible participants were identified (setting, location and dates)	56 (52.8)	95 (89.6)	32 (91.4)	32 (85.7)	33 (91.7)
	**9**	Whether participants formed a consecutive, random or convenience series	44 (41.51)	99 (93.4)	35 (100.0)	34 (97.1)	30 (83.3)
Test methods	**10a**	Index test, in sufficient detail to allow replication	104 (98.1)	100 (94.3)	34 (97.1)	34 (97.1)	32 (88.9)
	**10b**	Reference standard, in sufficient detail to allow replication	106 (100)	98 (92.5)	34 (97.1)	34 (97.1)	31 (86.1)
	**11**	Rationale for choosing the reference standard (if alternatives exist)	20 (18.9)	91 (85.9)	34 (97.1)	526 (71.4)	32 (88.9)
	**12a**	Definition of and rationale for test positivity cut-offs or result categories of the index test, distinguishing pre-specified from exploratory	97 (91.5)	71 (67)	32 (91.4)	21 (60)	18 (50)
	**12b**	Definition of and rationale for test positivity cut-offs or result categories of the reference standard, distinguishing pre-specified from exploratory	98 (92.5)	98 (92.5)	32 (91.4)	34 (97.1)	32 (88.9)
	**13a**	Whether clinical information and reference standard results were available to the performers/readers of the index test	Not applicable				
	**13b**	Whether clinical information and index test results were available to the assessors of the reference standard	29 (27.4)	91 (85.9)	33 (94.29)	25 (71.4)	33 (91.7)
Analysis	**14**	Methods for estimating or comparing measures of diagnostic accuracy	105 (99.1)	103 (97.2)	35 (100.00)	34 (97.1)	34 (94.4)
	**15**	How indeterminate index test or reference standard results were handled	93 (87.8)	93 (87.7)	35 (100.00)	33 (94.3)	25 (69.4)
	**16**	How missing data on the index test and reference standard were handled	62 (58.5)	66 (62.3)	33 (94.29)	26 (74.3)	7 (19.4)
	**17**	Any analyses of variability in diagnostic accuracy, distinguishing pre-specified from exploratory	27 (25.5)	85 (80.2)	33 (94.29)	21 (60)	31 (86.1)
	**18**	Intended sample size and how it was determined	6 (5.7)	104 (98.1)	35 (100)	34 (97.1)	35 (97.2)
**RESULTS**							
Participants	**19**	Flow of participants, using a diagram	0 (0)	104 (98.1)	35 (100)	33 (94.3)	37 (100.00)
	**20**	Baseline demographic and clinical characteristics of participants	28 (26.4)	92 (86.8)	34 (97.1)	39 (82.9)	29 (80.6)
	**21a**	Distribution of severity of disease in those with the target condition	105 (99.1)	97 (91.5)	34 (97.1)	33 (94.3)	30 (83.3)
	**21b**	Distribution of alternative diagnoses in those without the target condition	36 (34)	100 (94.3)	34 (97.1)	33 (94.3)	33 (91.7)
	**22**	Time interval and any clinical interventions between index test and reference standard	49 (46.2)	79 (74.5)	33 (94.3)	28 (80)	18 (50
Test results	**23**	Cross tabulation of the index test results (or their distribution) by the results of the reference standard	106 (100)	103 (97.2)	35 (100)	34 (97.1)	34 (94.4)
	**24**	Estimates of diagnostic accuracy and their precision (such as 95% confidence intervals)	89 (84)	88 (83)	33 (94.3)	28 (80)	27 (75)
**DISCUSSION**							
	**25**	Any adverse events from performing the index test or the reference standard	Not applicable				
	**26**	Study limitations, including sources of potential bias, statistical uncertainty, and generalisability	80 (75.5)	94 (88.68)	34 (97.1)	32 (91.4)	26 (77.8)
	**27**	Implications for practice, including the intended use and clinical role of the index test	34 (32.1)	71 (67)	35 (100.00)	24 (65.7)	13 (36.1)
**OTHER INFORMATION**							
	**28**	Registration number and name of registry	2 (1.9)	105 (99.1)	35 (100.00)	34 (97.1)	37 (100.00)
	**29**	Where the full study protocol can be accessed	2 (1.9)	105 (99.1)	35 (100.00)	34 (97.1)	37 (100.00)
	**30**	Sources of funding and other support; role of funders	23 (21.9)	86 (81.1)	35 (100.00)	27 (77.1)	25 (69.4)

Agreement with the senior rater differed for each of the three junior raters. The ophthalmology resident who had received introductory DTA research training agreed on all 31 items in more than 90% of the studies; agreement was almost perfect (97% to 100%) for 21 items (68%). Agreement with the clinical rater was variable (60% to 100%) but reached 94% or more for 17 items (54.8%). Agreement was poorer between the senior rater and the pharmacology researcher, who was experienced in intervention reviews but not in DTA reviews, as it was 20% to 83% in 13 items (41.9%) and was 86% to 100% in the others.

The kappa coefficient was 0.73 for 3286 assessments of the senior rater vs any other rater. When overall agreement was computed separately by the junior raters, kappa was 0.93 for the trained resident, 0.70 for the ophthalmologist and 0.55 for the pharmacology researcher. In a mixed logistic model with disagreement as a response variable and rater and item as random effects, the variance at the rater level was about 2/3 of that at the item level, suggesting a strong item-effect on disagreement. We investigated impact factor and publication year as potential determinants of disagreement. Study reports in higher impact journals led to less disagreement, but the effect was not statistically significant (p = 0.684 for the lowest vs highest tertiles). No effect of publication year was detected (p = 0.932 for linear trend).

### Effect of STARD adherence on QUADAS 2 assessment

We considered that specific STARD domains may influence the ability to rate signaling questions that guide the judgement on QUADAS 2 risk of bias domains, as explained in the Methods.

QUADAS 2 Patient Selection risk of bias domain was unclear in only 2 out of 106 studies, meaning that enough details were available to adjudicate low or high risk of bias in 98.1% of the studies. Despite this, STARD 2015 adherence was optimal only for item 6 (eligibility criteria: 97%), whereas items 7, 8 and 9 (overall concerning prior testing, setting and sampling) were reported in 41% to 52% of the studies. Moreover, items 20, 21a and 21b (patients’ characteristics, spectrum of glaucoma and alternative diagnoses, e.g. other types of ONH damage cause) were also variably reported since they were adherent in 27%, 99% and 35% of the studies respectively.

The Index Test domain of QUADAS 2 was unclear in 13 studies, thus risk of bias could be assessed in 87.7% of the studies as low risk (n. 65) or high risk (n. 28). Consistently, STARD 2015 items 10a and 12a (reporting on index test execution and threshold selection; item 13a concerning masking was not used for our objective tests) were reported in 89% and 93% of studies.

The Reference Standard domain of QUADAS 2 was unclear in 8 studies, thus risk of bias could be assessed in 92.5% of the studies (low risk in 97 and high risk in 1 study). The related STARD 2015 items were variably reported: items 10b and 12b (reporting on reference standard execution and threshold selection) were adherent in 99% and 100% of the studies, while items 11 and 13a (reference standard choice rationale and masking of reference standard to index test results) were reported in 21% and 30% of the studies.

The Flow and Timing domain of QUADAS 2 was unclear in 44 studies, thus risk of bias could be assessed in 58.5% of the studies (low risk in 15 and high risk in 47 studies). A patient flow diagram was not available in any study (nil adherence with item 19), while 55% of the studies fulfilled the requirement of item 22 since the interval between the index and reference tests was reported.

We did not consider QUADAS 2 domains of Applicability since the Patient selection applicability domain replicated the risk of bias grading, and the Index test and Reference standard applicability domains were no concern for nearly all the studies.

### Patterns of non-agreement

As mentioned above the raw agreement between the senior rater and any other rater was good overall. Table 2 presents comments on the potential causes of high or low agreement considering the adherence in reporting specific STARD 2015 items.

**Table 2:** Comments on main patterns of agreement or disagreement for each STARD 2015 item. Positive Reporting and Overall agreement, as presented in Table 1, are also shown for clarity.

	No	Item	Positive Reporting, N (%)	Overall agreement (%)	Comment
**TITLE OR ABSTRACT**					
	**1**	Identification as a study of diagnostic accuracy using at least one measure of accuracy (such as sensitivity, specificity, predictive values, or AUC)	106 (100)	101 (95.3)	Terms sensitivity, specificity or AUC always used in title or abstract
	**2**	Structured summary of study design, methods, results, and conclusions (for specific guidance, see STARD for abstracts)			Not applicable
**INTRODUCTION**					
	**3**	Scientific and clinical background, including the intended use and clinical role of the index test	10(9.4)	67(63.21)	Intended used of the test and the potential role in the clinical pathway often lacking or not clearly reported, thus difficult to assess
	**4**	Study objectives and hypotheses	40(37.4)	87(82.08)	Study objectives always reported but study hypothesis often lacking or not clearly reported
**METHODS**					
Study design	**5**	Whether data collection was planned before the index test and reference standard were performed (prospective study) or after (retrospective study)	59(55.7)	89(84)	Clear definition of prospective or retrospective nature of the study not always reported
Participants	**6**	Eligibility criteria	103(97.2)	97(91.5)	Generally well reported both for cases and controls
	**7**	On what basis potentially eligible participants were identified (such as symptoms, results from previous tests, inclusion in registry)	53(50)	77(72.6)	Details not always clearly reported for both cases and controls, thus difficult to assess
	**8**	Where and when potentially eligible participants were identified (setting, location and dates)	56(52.8)	95(89.6)	Date more often missing than setting
	**9**	Whether participants formed a consecutive, random or convenience series	44(41.51)	99(93.4)	Most studies had case-control design
Test methods	**10a**	Index test, in sufficient detail to allow replication	104(98.1)	100(94.3)	Characteristics of index test always reported and easy to retrieve
	**10b**	Reference standard, in sufficient detail to allow replication	106(100)	98(92.5)	Reference standard (visual field or optic nerve head appearance or both) always reported and easy to retrieve
	**11**	Rationale for choosing the reference standard (if alternatives exist)	20(18.9)	91(85.9)	Acknowledgment of incorporation bias made only in few cases
	**12a**	Definition of and rationale for test positivity cut-offs or result categories of the index test, distinguishing pre-specified from exploratory	97(91.5)	71(67)	The use of a large number of continuous and/or categorical parameters led to relatively low agreement among reviewers
	**12b**	Definition of and rationale for test positivity cut-offs or result categories of the reference standard, distinguishing pre-specified from exploratory	98(92.5)	98(92.5)	Clear definition of reference test criteria both for visual field and optic nerve head appearance
	**13a**	Whether clinical information and reference standard results were available to the performers/readers of the index test	Not applicable		
	**13b**	Whether clinical information and index test results were available to the assessors of the reference standard	29 (27.4)	91 (85.9)	Relatively easy to assess despite low adherence
Analysis	**14**	Methods for estimating or comparing measures of diagnostic accuracy	105 (99.1)	103 (97.2)	Easy to detect which measures of diagnostic accuracy were used
	**15**	How indeterminate index test or reference standard results were handled	93 (87.8)	93 (87.7)	Often stated that low quality images were not included in the analysis, but the item may have been interpreted differently
	**16**	How missing data on the index test and reference standard were handled	62 (58.5)	66 (62.3)	Comparison between number of enrolled and number of included patients in the final analysis was often needed to ascertain the existence of missing data
	**17**	Any analyses of variability in diagnostic accuracy, distinguishing pre-specified from exploratory	27 (25.5)	85 (80.2)	Low adherence, in most cases sub-analysis related to the disc size or disease severity among patients
	**18**	Intended sample size and how it was determined	6 (5.7)	104 (98.1)	Low adherence but easy to assess
**RESULTS**					
Participants	**19**	Flow of participants, using a diagram	0 (0)	104 (98.1)	Never reported
	**20**	Baseline demographic and clinical characteristics of participants	28 (26.4)	92 (86.8)	Age was almost always reported while sex, refraction and IOP were more often missing, but easy to assess
	**21a**	Distribution of severity of disease in those with the target condition	105 (99.1)	97 (91.5)	High adherence regarding glaucoma severity based on any classification system or mean deviation
	**21b**	Distribution of alternative diagnoses in those without the target condition	36 (34)	100 (94.3)	IOP in control patients often missing but easy to assess
	**22**	Time interval and any clinical interventions between index test and reference standard	49 (46.2)	79 (74.5)	Incompletely reported in the methods, results or discussion
Test results	**23**	Cross tabulation of the index test results (or their distribution) by the results of the reference standard	106 (100)	103 (97.2)	Never reported as 2X2 table but always derived from sensitivity/specificity data
	**24**	Estimates of diagnostic accuracy and their precision (such as 95% confidence intervals)	89 (84)	88 (83)	Estimates always reported but measures of precision sometimes missing
**DISCUSSION**					
	25	Any adverse events from performing the index test or the reference standard	Not applicable		
	26	Study limitations, including sources of potential bias, statistical uncertainty, and generalisability	80 (75.5)	94 (88.68)	At least one limitation often reported, mainly case control design or poor generalizability of the results due to the characteristics of included patients (disease severity or ethnicity)
	27	Implications for practice, including the intended use and clinical role of the index test	34 (32.1)	71 (67)	When reported, the pre-post test probability change or likelihood ratios were presented, rather than a discussion of false positive and false negative consequences
**OTHER INFORMATION**					
	28	Registration number and name of registry	2 (1.9)	105 (99.1)	Low adherence and high agreement, easy to assess
	29	Where the full study protocol can be accessed	2 (1.9)	105 (99.1)	Low adherence and high agreement, easy to assess
	30	Sources of funding and other support; role of funders	23 (21.9)	86 (81.1)	Low adherence and high agreement, easy to assess

Some items (1, 10a, 14, 18, 19, 23, 28, 29) showed very high agreement among raters due to the fact that they are less “subjectively” assessed as they rely on well-defined items, such as the use of technical terms (sensitivity, specificity, AUC) or objects (patient flow diagram).

Agreement may have been achieved more easily for items with almost perfect or absent adherence (n. 1, 6, 10a, 10b, 23, 28, 29), leading the rater to be driven towards always positive or always negative statements.

Of note, the Scientific and clinical background item showed modest agreement (63%) among raters. The evaluation of this item was affected by lack of clarity in reporting the clinical pathway in which the index test was to be used. In fact, authors often mentioned as the rationale for their study the ability of the test to detect the target disease, but the intended use of the index test (i.e. diagnosis, screening or monitoring) as well as its role in the clinical pathway were reported less clearly and were quite difficult to assess.

Similarly, there was modest inter-rater agreement on the reporting of the description of how potentially eligible participants were identified and included. The reporting and the assessment of such key features of a DTA study are not yet supported by detailed guidance that can be applied to specialized clinical research contexts [19]. Modest agreement was also observed for item 27 ‘Implications for practice’, which we feel should at least include the consequences of false positives and false negatives in the clinical pathway.

The definition of the test positivity cut-off showed modest agreement for the index test (item 12a, 67%) versus good agreement for the reference standard (item 12b, 92%). Specifically, the reference standard verification criteria were often clearly reported, while several continuous and categorical parameters were extracted for the index imaging tests, and a pre-specified positivity criterion to be used in analyses was less often presented. Moreover, our raters gave a different interpretation to analyses at fixed specificity (i.e. 0.95), which our guidance had suggested to be acceptable as threshold pre-specification.

Rating how missing data were handled showed a low level of raw agreement (62%). Unlike the reporting of how indeterminate results were handled, which was often clearly described as the exclusion of low image quality results, handling of missing data was difficult to assess. In fact, authors rarely specified the existence (or not) of missing data, and in most cases the number of missing data was derived by comparing the number of enrolled patients with those included in the final analysis.

## Discussion

Our study confirms previous findings of good reproducibility in assessing the quality of reporting of diagnostic accuracy studies using the original STARD statement. Smidt at al [20] found that disagreements were not so much caused by differences in interpretation of the items by the reviewers, but rather by difficulties in assessing the reporting of these items due to lack of clarity within the articles. In 2015, Fidalgo et al [14] investigated the use of STARD and QUADAS in 58 studies on automated perimetry for glaucoma and reported suboptimal reporting, with no improvement between 1993–2004 and 2004–2013. They inter-rater agreement (kappa) in their analyses was 0.70 and 0.81 for STARD and QUADAS respectively.

We observed a substantially higher agreement of the senior rater with the ophthalmology resident who had being trained on DTA systematic reviews as compared to the glaucoma specialist with limited DTA methodological training or the pharmacology researcher with good experience in intervention reviews, but no specific DTA and clinical training beyond the pilot assessment of 5 studies. The four raters acted independently, since they had not been working together before in DTA research. This stresses the importance of having both a clinical and a methodological background when undertaking DTA systematic reviews and related research. Piloting a few studies at the beginning of the review may not be sufficient.

Identifying the patterns of agreement/disagreement was one of the aims of our study. As could be expected, items which required judgement were more prone to “subjective” evaluation, such as the scientific and clinical background description or the reporting of implications for practice. These items showed lower agreement among reviewers due to difficulties in their assessment. Otherwise, items based on less “subjective” judgement, such as identification as a study of diagnostic accuracy based on measures of accuracy or a flow diagram, showed almost perfect agreement. This also implies that authors should increase their efforts in reporting these key points of their study, which are often more difficult to appraise.

We connected the completeness of reporting, assessed with STARD 2015, with methodological quality, evaluated with the help QUADAS 2, to investigate whether the former is a pre-requisite for the latter. However, we found it difficult to formally investigate this field, since several STARD 2015 items that were thought to relate to a given QUADAS 2 domain were variably adherent. Specifically, the QUADAS 2 domain “Patient Selection “was judged to be at low risk of bias for almost all studies, a finding that apparently is at odds with the observation that the corresponding STARD 2015 items were variably adherent, from about a quarter to nearly all studies. This suggests that context knowledge is additionally used to make decisions on risk of bias, and this goes beyond the transparency of reporting in the manuscript.

Our study has limitations, mainly due to very specific features of this research and clinical field. First, a single type of medical test was chosen in this study, i.e. imaging devices for diagnosing manifest glaucoma, and there may be unknown differences across medical specialties. Second, almost all studies included two groups of patients, cases and (healthy) controls. This led to rate QUADAS 2 Patient Selection domain as high risk of bias for most studies, which may have influenced the raters’ judgement by restricting uncertainty. Finally, the senior rater, but not the other raters, was aware of QUADAS 2 rating of all the studies when using STARD 2015, since he was the lead author of the related Cochrane review.

## Conclusions

STARD was developed to facilitate complete, informative and transparent reporting of diagnostic research, both by study authors, when they prepare a report, and by reviewers and readers, when they analyze the study report. We have shown that good reproducibility can be achieved when evaluating the reports of diagnostic accuracy studies with the STARD checklist, provided raters have sufficient prior training or experience in appraising diagnostic research. This finding reinforces the usability of STARD 2015 and should help its further dissemination and implementation.

## Supporting information

S1 Table Excel data set underlying the results described in the manuscript. (XLSX)
